# Decreased expression of RNA-binding motif protein 3 correlates with tumour progression and poor prognosis in urothelial bladder cancer

**DOI:** 10.1186/1471-2490-13-17

**Published:** 2013-04-08

**Authors:** Karolina Boman, Ulrika Segersten, Göran Ahlgren, Jakob Eberhard, Mathias Uhlén, Karin Jirström, Per-Uno Malmström

**Affiliations:** 1 Department of Clinical Sciences, Division of Pathology, Lund University, Skåne University Hospital, Lund, 221 85, Sweden; 2 Department of Surgical Sciences, Uppsala University, Uppsala, 751 85, Sweden; 3 Department of Clinical Sciences, Division of Urological Cancers, Lund University, Skåne University Hospital, Malmö, 205 02, Sweden; 4 Department of Clinical Sciences, Division of Oncology, Lund University, Skåne University Hospital, Lund, 221 85, Sweden; 5 Science for Life Laboratory and School of Biotechnology, AlbaNova University Center, Royal Institute of Technology, Stockholm, 106 91, Sweden; 6 School of Biotechnology, AlbaNova University Center, Royal Institute of Technology, Stockholm, 106 91, Sweden

**Keywords:** RBM3, Urothelial bladder cancer, Prognosis

## Abstract

**Background:**

Low nuclear expression of the RNA-binding motif protein 3 (RBM3) has previously been found to be associated with poor prognosis in several cancer forms e.g. breast, ovarian, colorectal, prostate cancer and malignant melanoma. The aim of this study was to examine the prognostic impact of RBM3 expression in urinary bladder cancer.

**Methods:**

Immunohistochemical RBM3 expression was examined in tumours from 343 patients with urothelial bladder cancer. Chi-square and Spearman’s correlation tests were applied to explore associations between RBM3 expression and clinicopathological characteristics. The impact of RBM3 expression on disease-specific survival (DSS), 5-year overall survival (OS) and progression-free survival (PFS) was assessed by Kaplan-Meier analysis and Cox proportional hazards modelling.

**Results:**

Reduced nuclear RBM3 expression was significantly associated with more advanced tumour (T) stage (p <0.001) and high grade tumours (p=0.004). Negative RBM3 expression was associated with a significantly shorter DSS (HR=2.55; 95% CI 1.68-3.86)) and 5-year OS (HR=2.10; 95% CI 1.56-2.82), also in multivariable analysis (HR=1.65; 95% CI 1.07-2.53 for DSS and HR=1.54; 95% CI 1.13-2.10 for 5-year OS). In patients with Ta and T1 tumours expressing reduced RBM3 levels, Kaplan-Meier analysis revealed a significantly shorter PFS (p=0.048) and 5-year OS (p=0.006).

**Conclusion:**

Loss of RBM3 expression is associated with clinically more aggressive tumours and an independent factor of poor prognosis in patients with urothelial bladder cancer and a potentially useful biomarker for treatment stratification and surveillance of disease progression.

## Background

Approximately 20% of patients with urothelial carcinoma of the bladder present with muscle invasive cancer [1]. However, the majority of bladder malignancies do not invade muscle at diagnosis (Tis, Ta, and T1). The clinical problem associated with these tumors is their highly unpredictable potential for recurrence and progression into muscle invasive disease [1]. High-grade bladder tumors with lamina propria invasion (T1) represent those at the greatest risk, rendering the surgical management of this disease subject to much controversy [2]. Nearly one-third of these patients will require cystectomy as a second-line treatment after failure of Bacillus Calmette-Guerin (BCG) treatment [3]. The challenge is to identify these high-risk cases upfront, to offer them cystectomy as primary treatment. For patients with muscle-invasive bladder cancer, cystectomy with pelvic lymph node dissection remains the mainstay of treatment. Overall, approximately 50% of these patients will develop distant metastases after surgical treatment and die of the disease [4]. The prognosis worsens for patients with tumours involving perivesical fat or adjacent organs (cT3b-4) and those with lymph node involvement. In these patients, cystectomy alone offers a cure rate of only 20%–30% [5]. Hence, there is a great need for novel prognostic and treatment predictive biomarkers to improve clinical management of patients with urothelial bladder cancer.

Reduced expression of the RNA-binding motif protein 3 (RBM3) has previously been demonstrated to correlate with an impaired prognosis in several major human cancer forms i.e. breast, ovarian, prostate, colorectal cancer and malignant melanoma [6-10]. While the functional basis for these observations remain to be fully elucidated, the observed association between RBM3 expression and DNA integrity and repair [10,11] may be of importance.

In the present study, we examined the prognostic significance of RBM3 expression in tumours from a large prospective cohort of patients with urothelial bladder cancer.

## Methods

### Patients

All patients with newly diagnosed urothelial bladder cancer at Uppsala University Hospital have been registered prospectively since 1984. This study included patients diagnosed up until 2005 for whom histological specimens were available. Since the majority of tumours was made up of Ta tumours, this group was reduced to include 115 tumours. Patient and tumour characteristics are summarised in Table 1. Progression-free survival (PFS), overall survival (OS) and disease-specific survival (DSS) were calculated from the date of surgery to date of event or last follow-up. At follow up, patients with non-muscle invasive tumours were categorized as having none, few or frequent recurrences. Definition of few recurrences was less than three recurrent tumours within 18 months, whereas frequent recurrences were three or more recurrences within the same time period. Progression was defined as shift of the tumor into a higher stage. Median time to progression for patients with non-muscle invasive disease was 18.0 months (range 2.0-55.0). Follow-up time for non-recurrent and non-progressing cases were ≥4 and ≥5-years, respectively.

**Table 1:** Distribution of patient and tumour characteristics in the evaluated cohort (n=343)

	
**Age**	
Mean	71.85
Median	73.00
(Range)	31-96
**Sex**	
Female	83 (24.2)
Male	260 (75.8)
**Smoking**	
Smoker	130 (79.8)
Non-smoker	33 (20.2)
*Unknown*	180
**T-stage**	
pTa	115 (33.5)
pT1	116 (33.8)
pT2	90 (26.2)
pT3	17 (1.5)
pT4	5 (5.0)
**Grade**	
Low	82 (23.9)
High	261(76.1)
**N-stage**	
0	43 (78.2)
1	12 (21.8)
unknown	288
**M-stage**	
0	92 (70.2)
1	39 (29.8)
unknown	212

### Tissue microarray construction

The use of these patient samples for protein profiling was approved by the regional ethical review board of Uppsala (reference number 2005:339). All tumours were histopathologically re-evaluated and classified according to the WHO grading system of 2004 [12] by a board certified pathologist. Tissue microarrays (TMAs) were constructed using a semi-automated arraying device (TMArrayer, Pathology Devices, Westminister, MD, USA). All tumour samples were represented in duplicate tissue cores (1mm).

### Immunohistochemistry and staining evaluation

For immunohistochemical analysis, 4 μm TMA-sections were automatically pre-treated using the PT Link system and then stained in an Autostainer Plus (DAKO; Glostrup, Copenhagen, Denmark) with the mouse monoclonal anti-RBM3 antibody AAb030038 (Atlas Antibodies AB, Stockholm, Sweden) diluted 1:10000. The specificity of the antibody has been validated previously [6,10], also in the human bladder cancer cell line RT-4, in which RBM3 was demonstrated to be highly expressed [6]. RBM3 staining was evaluated by two independent observers (KB and KJ) who were blinded to clinical and outcome data. RBM3 was mainly expressed in the nuclei and the fraction of cells with nuclear positivity (NF) denoted as 0 (0-1%), 1 (2-25%), 2 (26-75%), 3 (>75%), and the intensity of staining (NI) as 0 (negative), 1 (weak), 2 (moderate) and 3 (strong). The fraction of positively staining cells was estimated across both sampled cores, and the dominating staining intensity denoted. A combined nuclear score (NS), e.g. multiplier of NF × NI, was then constructed as previously described [9,10].

### Statistics

Spearman’s rho and Chi-square tests were applied for analysis of the correlation between RBM3 expression and clinicopathological characteristics. Kaplan-Meier analysis and log rank test were used to illustrate differences in progression free survival (PFS), disease-specific survival (DSS), overall survival (OS) and 5-year OS in strata according to RBM3 expression. For survival analyses, RBM3 expression was trichotomized into negative (NS=0), intermediate (NS=1-6) and high (NS=9), or dichotomised into negative (NS=0) vs positive (NS≥1) or negative-intermediate (NS 0–6) versus high (NS=9). Cox regression proportional hazards modelling was used to estimate the impact of negative vs positive RBM3 expression on DSS, 5-year OS, PFS in both univariable and multivariable analysis, adjusted for age, sex, T-stage and grade. All tests were two sided. A p-value of 0.05 was considered significant. All statistical analyses were performed using IBM SPSS Statistics version 20.0 (SPSS Inc., Chicago, IL, USA).

## Results

### Associations between RBM3 expression, patient and tumour characteristics

**Figure 1:**
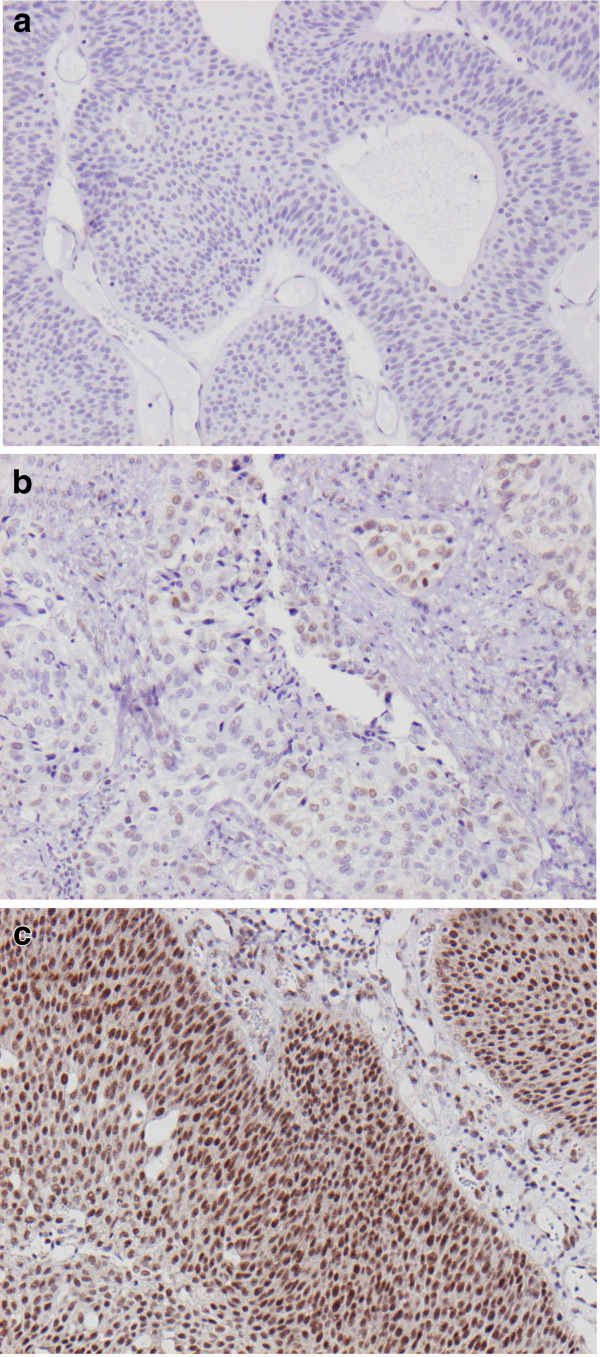
**Sample immunohistochemical images of RBM 3 staining in urothelial bladder cancer.** Images (20x magnification) representing (**a**) negative, (**b**) intermediate and (**c**) high nuclear RBM3 expression.

Following antibody optimisation and staining, RBM3 expression could be evaluated in tumours from 343/344 (99,7%) cases. There was no obvious heterogeneity in RBM3 expression between duplicate TMA cores. Negative RBM3 staining (NS=0) was denoted in 77 (22.4%) cases, weak-strong intensity in <75% of the cells (NS 1–6) in 213 (62.1%) cases and strong staining in > 75% of the cells (NS=9) was denoted in 53 (15.5%) cases. Heterogeneity regarding the nuclear staining fraction between duplicate cores was only observed in some cases with intermediate RBM3 expression, and never exceeding 1 category. Images representing different patterns of RBM expression are shown in Figure 1a-c.

We examined the relationship between three categories of RBM3 expression; negative (NS=0), intermediate (NS=1-6) and high (NS=9), and established clinicopathological parameters (Table 2). This revealed a significant positive association between RBM3 expression and a younger age at diagnosis (p=0.013), lower T-stage (p<0.001) and high grade tumours (p=0.004).

**Table 2:** Associations between RBM3 expression and clinicopathological characteristics.

**RBM3 expression**	**Negative (NS=0)**	**Intermediate (NS=1-6)**	**High(NS=9)**	
n(%)	77 (22.4)	213 (62.1)	53 (15.5)	*p-value*
**Age**				
≤ average	24 (31.2)	107 (50.2)	27 (50.9)	0.013
>average	53 (68.8)	106 (59.8)	26 (49.1)	
**Gender**				
Female	26 (33.8)	45 (21.1)	12 (22.6)	0.092
Male	51 (66.2)	168 (78.9)	41 (77.4)	
**T-stage**				
Ta	18 (23.4)	74 (34.7)	23 (43.4)	<0.001
T1	15 (19.5)	77 (36.2)	24 (45.3)	
T2-4	44 (57.1)	62 (29.1)	6 (11.3)	
**Grade**				
Low	8 (10.4%)	58 (27.2)	16 (30.2)	0.004
High	69 (89.6%)	155 (72.8)	37 (69.8)	

### Association between RBM3 expression and survival in the full cohort

**Figure 2:**
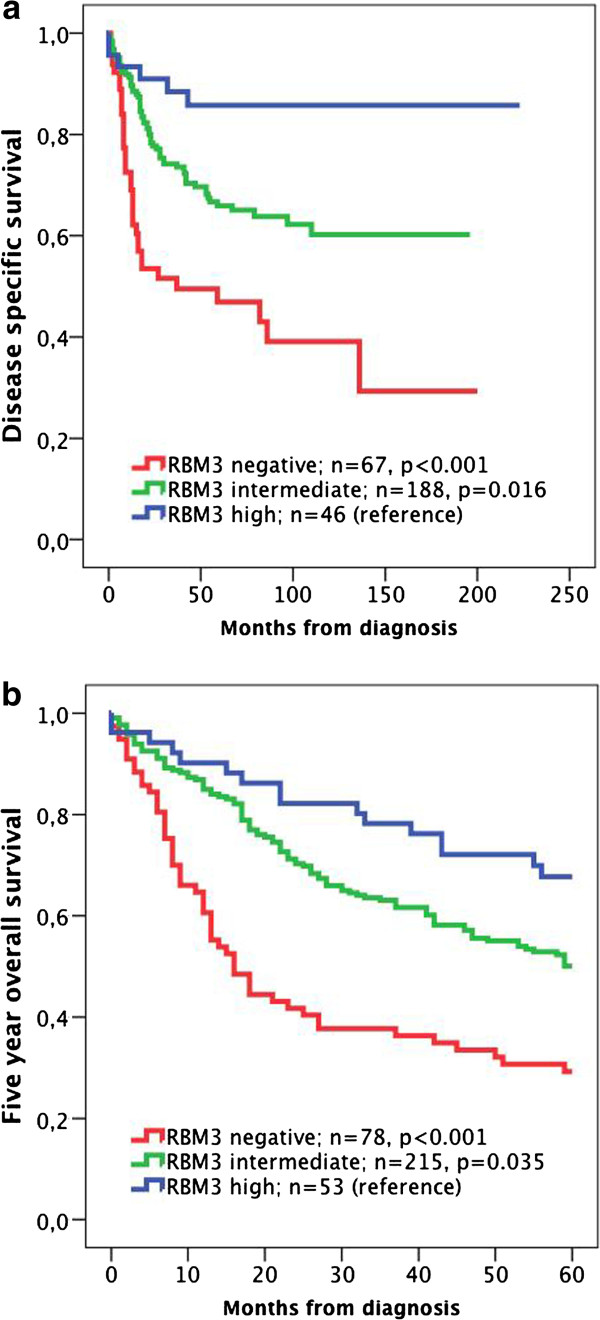
**Kaplan-Meier estimates of bladder cancer specific survival and 5-year overall survival in the full cohort.** Kaplan-Meier analysis of (**a**) bladder cancer specific and (**b**) overall survival in strata of negative, intermediate and high RBM3 expression.

Kaplan-Meier analysis and log-rank test revealed a stepwise reduced DSS (Figure 2a) and 5-year OS (Figure 2b) with decreasing RBM3 expression, with the shortest DSS (p<0.001) and 5-year OS (p<0.001) for patients with tumours lacking RBM3 expression. These associations were confirmed in Cox univariable analysis (HR=2.55; 95% CI 1.68-3.86 for DSS and HR=2.10; 95% CI 1.56-2.82 for 5-year OS) and remained significant in multivariable analysis adjusted for age, gender, T-stage and grade (HR=1.65; 95% CI 1.07-2.53 for DSS and HR=1.54; 95% CI 1.13-2.10 for 5-year OS) (Table 3). Separate analysis of categories of nuclear fraction and staining intensity yielded similar results for DSS and 5-year OS (Additional file 1).

**Table 3:** Relative risks of death from disease and overall death within 5 years according to clinicopathological factors and RBM3 expression in all patients (n=343)

		**Risk of death from disease**			**Risk of death within 5 years**	
		**Univariable**	**Multivariable**		**Univariable**	**Multivariable**
	**n (events)**	**HR (95% CI)**	**HR (95% CI)**	**n (events)**	**HR (95% CI)**	**HR (95% CI)**
**Age**						
Continuous	299 (100)	1.05 (1.03-1.07)	1.04 (1.02-1.07)	343 (123)	1.07 (1.05-1.08)	1.07 (1.05-1.08)
**Gender**						
Female	72 (28)	1.00	1.00	83 (56)	1.00	1.00
Male	227 (72)	0.79 (0.51-1.23)	1.07 (0.68-1.67)	260 (167)	0.95 (0.70-1.29)	1.24 (0.91-1.70)
**Stage**						
Ta	104 (13)	1.00	1.00	115 (61)	1,00	1.00
T1	97 (25)	2.20 (1.13-4.31)	1.87 (0.85-4.08)	116 (71)	1.37 (0.97-1.94)	1.44 (0.96-2.15)
T2-4	99 (62)	8.86 (4.86-16.16)	6.77 (3.21-14.26)	112 (91)	2.79 (2.01-3.87)	2.90 (1.92-4.40)
**Grade**						
Low	75 (7)	1.00	1.00	82 (41)	1.00	1.00
High	224 (93)	5.75 (2.66-12.40)	1.52 (1.58-3.96)	261 (182)	2.04 (1.45-2.87)	0.90 (0.58-1.39)
**RBM3 expression**						
Positive	232 (66)	1.00	1.00	266 (160)	1.00	1.00
Negative	67 (34)	2.55 (1.68-3.86)	1.65 (1.07-2.53)	77 (63)	2.10 (1.56-2.82)	1.54 (1.13—2.10)

### Association between RBM3 expression, disease progression and survival in patients with Ta and T1 tumours

**Figure 3:**
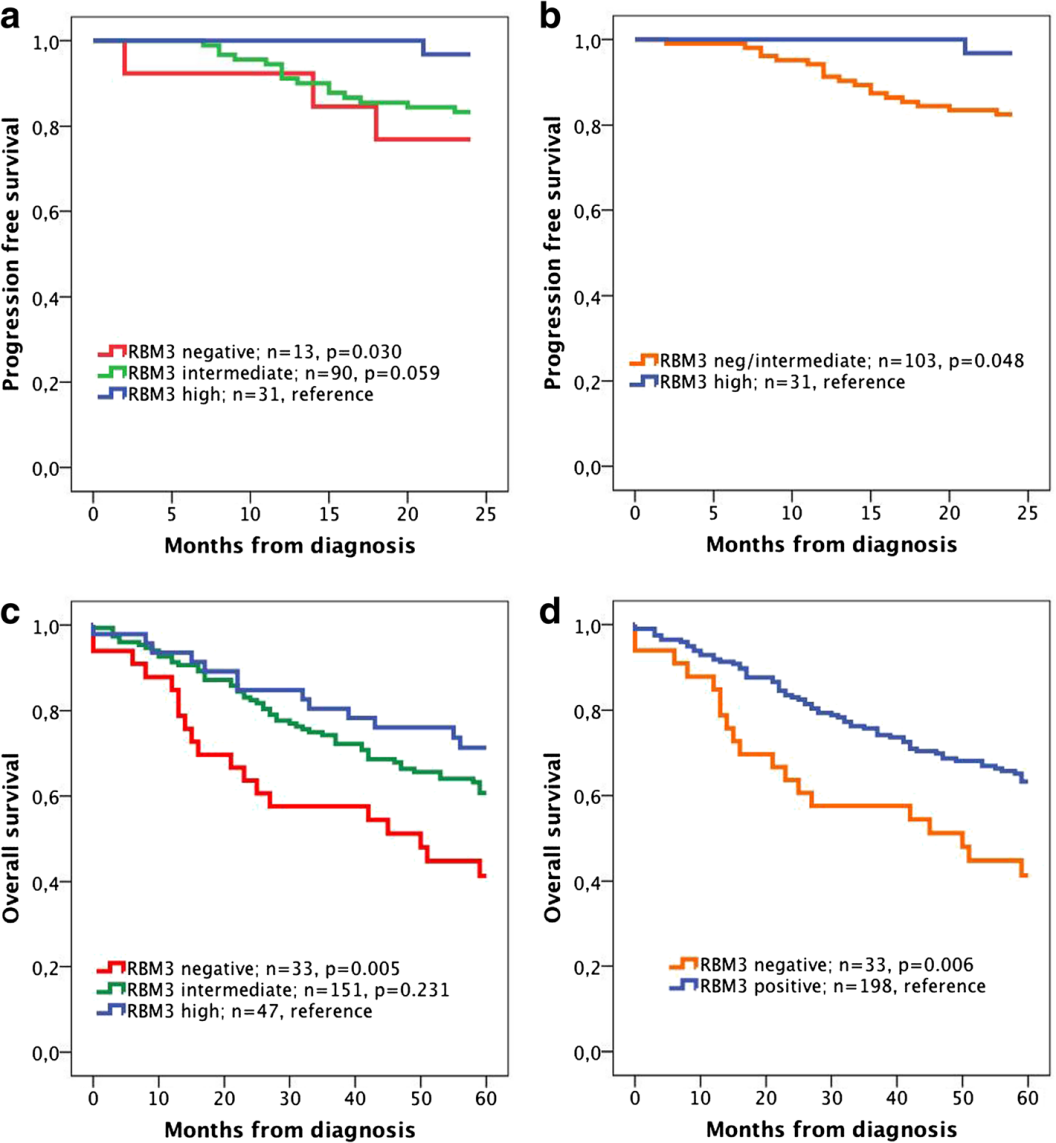
**Kaplan-Meier estimates of progression free survival and 5-year overall survival in patients with Ta and T1 tumours.** Kaplan-Meier analysis of progression free survival in strata according to (**a**) negative, intermediate and high RBM3 expression and (**b**) negative-intermediate versus high expression, and 5-year overall survival in (**c**) negative, intermediate and high RBM3 expression and (**d**) negative versus positive expression.

Next, we examined the association between RBM3 expression, disease progression within 24 months and 5-year OS in patients with Ta and T1 tumours. Kaplan-Meier analysis using three categories of RBM3 expression revealed a significantly reduced PFS for patients with RBM3 negative tumours compared to tumours with high RBM3 expression (p=0.030). A similarly reduced, borderline significant, PFS was demonstrated for tumours with intermediate RBM3 expression (Figure 3a), and when a dichotomised variable of high versus negative/intermediate RBM3 expression was applied, a significantly reduced PFS was seen for the latter category (p=0.048, Figure 3b). Analysis of 5-year OS using the trichotomised variable of RBM3 expression (Figure 3c) revealed a significantly reduced survival for patients with RBM3 negative tumours compared to those with high expression (p=0.005) while survival was similar for the intermediate category for RMB3 expression, as also demonstrated using a dichotomised variable comparing negative RBM3 expression with any RBM3 expression (p=0.006, Figure 3d).

Cox regression analysis demonstrated that neither RBM3 expression nor any of the established clinicopathological factors were significantly associated with PFS, but that grade and RBM3 expression (negative-intermediate vs high) were the best predictors of disease progression within 24 months in both univariable and multivariable analysis (Table 4). However, a significantly shorter 5-year OS was seen for RBM3 negative compared to RBM3 positive tumours in univariable analysis (n=231; HR=2.00 95% CI 1.21-3.33) and borderline significant in multivariable analysis (HR=1.64 95% CI 0.96-2.78) (Table 4). There was a borderline significant association between RMB3 expression and DSS in both univariable analysis (n=201; HR=3.06 95% CI 0.95-10.00) and multivariable analysis (n=201; HR=3.24 95% CI 1.00-10.53). There was no significant association between RBM3 expression and the rate of recurrence (none, few or frequent) (data not shown).

**Table 4:** Relative risks of progression within 24 months and overall death within 5 years according to clinicopathological factors and RBM3 expression in patients with Ta and T1 tumours

		**Risk of progression within 24 months**				**Risk of death within 5 years**	
		**Univariable**	**Multivariable**			**Univariable**	**Multivariable**
	**n(events)**	**HR(95%CI)**	**HR(95%CI)**		**n(events)**	**HR(95%CI)**	**HR(95%CI)**
**Age**							
Continuous	134(19)	1.02(0.98-1.07)	1.01(0.97-1.06)		231(88)	1.06(1.04-1.09)	1.06(1.04-1.08)
**Gender**							
Female	29(2)	1.00	1.00		43(12)	1.00	1.00
Male	105(17)	2.48(0.57-10.74)	1.98(0.45-8.64)		188(76)	0.62(0.34-1.13)	1.67(0.90-3.10)
**Grade**							
Low	48(3)	1.00	1.00		81(19)	1.00	1.00
High	86(16)	3.24(0.94-11.12)	3.32(0.97-11.40)		150(69)	2.22(1.33-3.68)	1.31(0.73-2.35)
**Stage**							
Ta	68(7)	1.00	1.00		115(35)	1.00	1.00
T1	66(12)	1.90(0.75-4.83)	1.24(0.44-3.46)		116(53)	0.61(0.40-0.93)	1.61(1.04-2.49)
RBM3 expression*				RBM3 expression*			
High	31(1)	1.00	1.00	Positive	198(69)	1.00	1.00
Negative/Intermediate	103(18)	5.92(0.79-44.39)	6.10(0.81-45.69)	Negative	33(19)	2.00(1.21-3.33)	1.64(0.96-2.78)

For PFS a dichotomised variable comparing patients with negative or intermediate RBM3 expression to those with high expression was applied. For 5 year OS, a dichotomised variable of negative versus any RBM3 expression was applied.

Similar findings were seen in subgroup analysis of patients with T1 tumours, where none of the patients with tumours expressing high levels of RMB3 (n=14) had disease progression during 24 months follow-up, in contrast to tumours with intermediate and negative expression (n= 52, 12 events) (data not shown). In patients with T1 tumours, a significant correlation was seen between negative RBM3 expression and a reduced 5-year OS in both univariable analysis (n=116; HR=2.44 95% CI 1.25-4.75) and multivariable analysis (HR=1.98 95% CI 1.01-3.90).

## Discussion

The results from this study show that reduced RBM3 expression is significantly associated with more aggressive tumours and an independent predictor of reduced survival in patients with urothelial bladder cancer. Moreover, in patients with Ta and T1 tumours, reduced RBM3 expression correlated with a significantly shorter time to disease progression, despite the comparatively low number of events, and negative RBM3 expression was an independent predictor of a reduced 5-year overall survival. The association between RBM3 expression and a favorable clinical outcome has been demonstrated in several other cancer forms [6-10] but this is, to our knowledge, the first report of the prognostic impact of RBM3 expression in urothelial bladder cancer.

In this comparatively large study of retrospectively collected tumours from a prospective cohort of patients with urothelial bladder cancer, the most evident impact of RBM3 expression was seen for disease specific and overall survival, both in the full cohort and in subgroup analysis of patients with Ta and T1 tumours. The fact that the impact on progression-free survival in patients with Ta and T1 tumours was not as significant may be due to the lower number of events, and therefore, the association between RBM3 expression and tumour progression merits further validation in other cohorts. Notably, RBM3 expression was not associated with neither occurrence nor frequency of local recurrence. These findings may indeed indicate different, or even opposing, tumour biological roles for RBM3 in tumour initiation and progression of urothelial bladder cancer. Previous studies, e.g. in malignant melanoma, have demonstrated that the correlation between reduced RBM3 expression and overall survival was more evident than the correlation to recurrent disease [8]. Speculatively, these findings could indicate that loss of RBM3 expression results in a tumour phenotype more prone to metastatic spread than local aggressiveness. RBM3 has previously been shown to be upregulated in neoplastic as compared to normal tissue [7,9], which also seems to be true for urothelial bladder epithelium versus urothelial neoplasms (http://www.proteinatlas.org). While urothelial bladder cancer is in its nature a recurrent disease [1], a recurrence does not per se affect survival from the disease. It is therefore plausible to assume that tumours with high RBM3 expression may well recur, while loss of RBM3 expression will generate a disease more likely to muscle invasion and distant spread.

The precise functional mechanisms behind loss of RBM3 and a more aggressive tumour behaviour remain to be elucidated. However, in vitro models need to be designed and interpreted with the impact of RBM3 expression on clinical outcome in mind, and the previously demonstrated pro-tumourigenic properties for RBM3 in vitro, e.g. that siRNA mediated silencing of the gene renders cancer cells less proliferative [10,13,14], should not be interpreted as being contradictory to its association with a prolonged survival when expressed in human tumours.

In a translational context, further probing of the association between RBM3 and DNA integrity and repair, that has been demonstrated in epithelial ovarian cancer in vivo and in vitro [11], may give more insight into the mechanistic basis for the favourable prognostic impact of RBM3 expression, once a tumour has been established. The link between RBM3 expression and an attenuated DNA damage response not only fits with its previously demonstrated association with cisplatin sensitivity , but also its association with good prognosis, since a deficient DNA repair system may well decrease the capability of invasion and metastatic spread [15,16]. Along this line, it would also be of interest to investigate the impact of RBM3 as a predictor of response to platinum-based chemotherapy, both in the neoadjuvant, adjuvant and palliative setting, cisplatin being one of the cornerstones in the medical treatment of bladder cancer [17-20]. Few molecular markers have proven to be efficient predictors of treament response, and none have been incorporated into clinical protocols. The question of the role of RBM3 as a marker for platinum sensitivity could not be addressed in the present study, as treatment data was lacking for the majority of the patients.

The clinical management of urothelial bladder cancer holds room for improvement. The monitoring is not only troublesome for the patients, but is also costly for the health care system. In fact, due to its high rate of recurrence and invasive monitoring requirements, urothelial bladder cancer has the highest lifetime treatment cost per patient of all cancers in the United States [21]. Hence, the benefit of finding tools to better identify patients more likely to have, or being at risk of developing, muscle invasive and metastatic desease is evident. Here, we have demonstrated a stepwise reduced survival with decreasing levels of nuclear RBM3 expression. While the greatest impact on DSS and OS was seen for patients with tumours lacking RBM3 expression, an increased risk of disease progression was also observed in the patient group with intermediate expression. This observation, together with the strong correlation between loss of RBM3 and muscle invasive disease, indicates that loss of RBM3 expression may indeed be a marker of disease progression. Therefore, immunohistochemical assessment of RBM3 expression could prove to be a valuable tool to more accurately predict muscle invasion, even in suboptimal samples from transurethral resections of the bladder. Moreover, it would be of interest to evaluate whether immunocytochemical RBM3 expression could be a useful diagnostic tool for urine cytology samples, to distinguish between different low-grade urothelial neoplastic lesions, e.g. papillary neoplasms of low malignant potential and low-grade papillary urothelial carcinomas, entities with considerable cytologic-histologic discrepancies [22]. While the findings in the present study are based on analyses of transitional cell carcinoma, it would also be of interest to examine RBM3 expression and its possible prognostic implications in other histological subtypes of bladder cancer, e.g. squamous cell carcinoma or adenocarcinoma.

## Conclusions

The results from this study suggest that immunohistochemical evaluation of RBM3 expression in urothelial bladder cancer specimens may be a valuable diagnostic and prognostic biomarker, and a useful tool for improved treatment stratification of patients suffering from the disease.

## Abbreviations

RBM3: RNA-binding motif protein 3; DSS: Disease-specific survival; OS: Overall survival; PFS: Progression-free survival, NS, Nuclear score; TMA: Tissue microarray; HR: Hazard ratio; CI: Confidence interval

## Competing interests

Jakob Eberhard and Karin Jirström hold pending intellectual property in relation to RBM3 as a prognostic biomarker in the treatment of bladder cancer. The remaining authors declare that they have no competing interests.

## Authors’ contributions

KB evaluated the immunohistochemical stainings, performed the statistical analyses and drafted the manuscript, US collected clinical data, assisted with the statistical analysis and helped draft the manuscript, GA assisted with the statistical analysis and helped draft the manuscript, JE contributed with the conception and design of the study, MU contributed with antibody validation, KJ conceived of the study, evaluated the immunohistochemistry, and helped draft the manuscript, PUM is responsible for the patient cohort and clinical database, and helped draft the manuscript. All authors read and approved the final manuscript.

## Pre-publication history

The pre-publication history for this paper can be accessed here:

http://www.biomedcentral.com/1471-2490/13/17/prepub

## Supplementary Material

Additional file 1 **Prognostic value of categories of nuclear fcation and intensity of RBM3 staining.** Kaplan-Meier analysis of (**A**, **B** bladder cancer specific and (**C**, **D**) overall survival according to the nuclear fraction and intensity of RBM3 staining, respectively (logrank p over strata <0.001 for all). (JPEG 1141 kb) Click here for file

## References

[B1] Raghavan D Shipley WU Garnick MB Russell PJ Richie JP Biology and management of bladder cancer N Engl J Med 1990 322 16 1129 1138 10.1056/NEJM199004193221607 2181313

[B2] Masood S Sriprasad S Palmer JH Mufti GR T1G3 bladder cancer–indications for early cystectomy Int Urol Nephrol 2004 36 1 41 44 15338671 10.1023/b:urol.0000032688.37789.7c

[B3] Lebret T Watson RW Molinie V Poulain JE O'Neill A Fitzpatrick JM Botto H HSP90 expression: a new predictive factor for BCG response in stage Ta-T1 grade 3 bladder tumours Eur Urol 2007 51 1 161 166 discussion 166–167 10.1016/j.eururo.2006.06.006 16828965

[B4] Sternberg CN The treatment of advanced bladder cancer Ann Oncol 1995 6 2 113 126 7786818 10.1093/oxfordjournals.annonc.a059105

[B5] Soloway MS Lopez AE Patel J Lu Y Results of radical cystectomy for transitional cell carcinoma of the bladder and the effect of chemotherapy Cancer 1994 73 7 1926 1931 10.1002/1097-0142(19940401)73:7<1926::AID-CNCR2820730725>3.0.CO;2-Q 8137219

[B6] Hjelm B Brennan DJ Zendehrokh N Eberhard J Nodin B Gaber A Ponten F Johannesson H Smaragdi K Frantz C High nuclear RBM3 expression is associated with an improved prognosis in colorectal cancer Proteomics Clin Appl 2011 5 11–12 624 635 21956899 10.1002/prca.201100020

[B7] Jogi A Brennan DJ Ryden L Magnusson K Ferno M Stal O Borgquist S Uhlen M Landberg G Pahlman S Nuclear expression of the RNA-binding protein RBM3 is associated with an improved clinical outcome in breast cancer Modern pathology: an official journal of the United States and Canadian Academy of Pathology, Inc 2009 22 12 1564 1574 10.1038/modpathol.2009.124 19734850

[B8] Jonsson L Bergman J Nodin B Manjer J Ponten F Uhlen M Jirstrom K Low RBM3 protein expression correlates with tumour progression and poor prognosis in malignant melanoma: an analysis of 215 cases from the Malmo Diet and Cancer Study J Transl Med 2011 9 114 10.1186/1479-5876-9-114 21777469 PMC3156749

[B9] Jonsson L Gaber A Ulmert D Uhlen M Bjartell A Jirstrom K High RBM3 expression in prostate cancer independently predicts a reduced risk of biochemical recurrence and disease progression Diagn Pathol 2011 6 91 10.1186/1746-1596-6-91 21955582 PMC3195697

[B10] Ehlen A Brennan DJ Nodin B O'Connor DP Eberhard J Alvarado-Kristensson M Jeffrey IB Manjer J Brandstedt J Uhlen M Expression of the RNA-binding protein RBM3 is associated with a favourable prognosis and cisplatin sensitivity in epithelial ovarian cancer J Transl Med 2010 8 78 10.1186/1479-5876-8-78 20727170 PMC2936876

[B11] Ehlen A Nodin B Rexhepaj E Brandstedt J Uhlen M Alvarado-Kristensson M Ponten F Brennan DJ Jirstrom K RBM3-regulated genes promote DNA integrity and affect clinical outcome in epithelial ovarian cancer Translational oncology 2011 4 4 212 221 21804916 10.1593/tlo.11106 PMC3140008

[B12] Sauter GAF Amin MB In Elbe JN EJ, Sesterhenn I Noninvasive urothelial neoplasias; WHO classification of noninvasive papillary urothelial tumours A World Health Organization Classification of Tumours Pathology and Genetics of Tumours of the Urinary System and Male Genital Organs 2004 Lyon: IARCC

[B13] Sureban SM Ramalingam S Natarajan G May R Subramaniam D Bishnupuri KS Morrison AR Dieckgraefe BK Brackett DJ Postier RG Translation regulatory factor RBM3 is a proto-oncogene that prevents mitotic catastrophe Oncogene 2008 27 33 4544 4556 10.1038/onc.2008.97 18427544 PMC2677646

[B14] Wellmann S Truss M Bruder E Tornillo L Zelmer A Seeger K Buhrer C The RNA-binding protein RBM3 is required for cell proliferation and protects against serum deprivation-induced cell death Pediatr Res 2010 67 1 35 41 10.1203/PDR.0b013e3181c13326 19770690

[B15] Bartkova J Rajpert-De Meyts E Skakkebaek NE Lukas J Bartek J DNA damage response in human testes and testicular germ cell tumours: biology and implications for therapy Int J Androl 2007 30 4 282 291 discussion 291 10.1111/j.1365-2605.2007.00772.x 17573848

[B16] Bartkova J Horejsi Z Koed K Kramer A Tort F Zieger K Guldberg P Sehested M Nesland JM Lukas C DNA damage response as a candidate anti-cancer barrier in early human tumorigenesis Nature 2005 434 7035 864 870 10.1038/nature03482 15829956

[B17] Loehrer PJ Sr Einhorn LH Elson PJ Crawford ED Kuebler P Tannock I Raghavan D Stuart-Harris R Sarosdy MF Lowe BA A randomized comparison of cisplatin alone or in combination with methotrexate, vinblastine, and doxorubicin in patients with metastatic urothelial carcinoma: a cooperative group study J Clin Oncol 1992 10 7 1066 1073 1607913 10.1200/JCO.1992.10.7.1066

[B18] Meeks JJ Bellmunt J Bochner BH Clarke NW Daneshmand S Galsky MD Hahn NM Lerner SP Mason M Powles T A systematic review of neoadjuvant and adjuvant chemotherapy for muscle-invasive bladder cancer Eur Urol 2012 62 3 523 533 10.1016/j.eururo.2012.05.048 22677572

[B19] Sternberg CN Bellmunt J Sonpavde G Siefker-Radtke AO Stadler WM Bajorin DF Dreicer R George DJ Milowsky MI Theodorescu D ICUD-EAU International Consultation on Bladder Cancer 2012: chemotherapy for urothelial carcinoma-neoadjuvant and adjuvant settings Eur Urol 2013 63 1 58 66 10.1016/j.eururo.2012.08.010 22917984

[B20] von der Maase H Hansen SW Roberts JT Dogliotti L Oliver T Moore MJ Bodrogi I Albers P Knuth A Lippert CM Gemcitabine and cisplatin versus methotrexate, vinblastine, doxorubicin, and cisplatin in advanced or metastatic bladder cancer: results of a large, randomized, multinational, multicenter, phase III study J Clin Oncol 2000 18 17 3068 3077 11001674 10.1200/JCO.2000.18.17.3068

[B21] Sievert KD Amend B Nagele U Schilling D Bedke J Horstmann M Hennenlotter J Kruck S Stenzl A Economic aspects of bladder cancer: what are the benefits and costs? World J Urol 2009 27 3 295 300 10.1007/s00345-009-0395-z 19271220 PMC2694315

[B22] Raab SS Grzybicki DM Vrbin CM Geisinger KR Urine cytology discrepancies: frequency, causes, and outcomes Am J Clin Pathol 2007 127 6 946 953 10.1309/XUVXFXMFPL7TELCE 17509992

