# Supplementary figures and images for: Decreased expression of RNA-binding motif protein 3 correlates with tumour progression and poor prognosis in urothelial bladder cancer
Source: BMC Urol. 2013 Apr 8;13:17. doi: 10.1186/1471-2490-13-17 (PMC3635919; doi:10.1186/1471-2490-13-17)

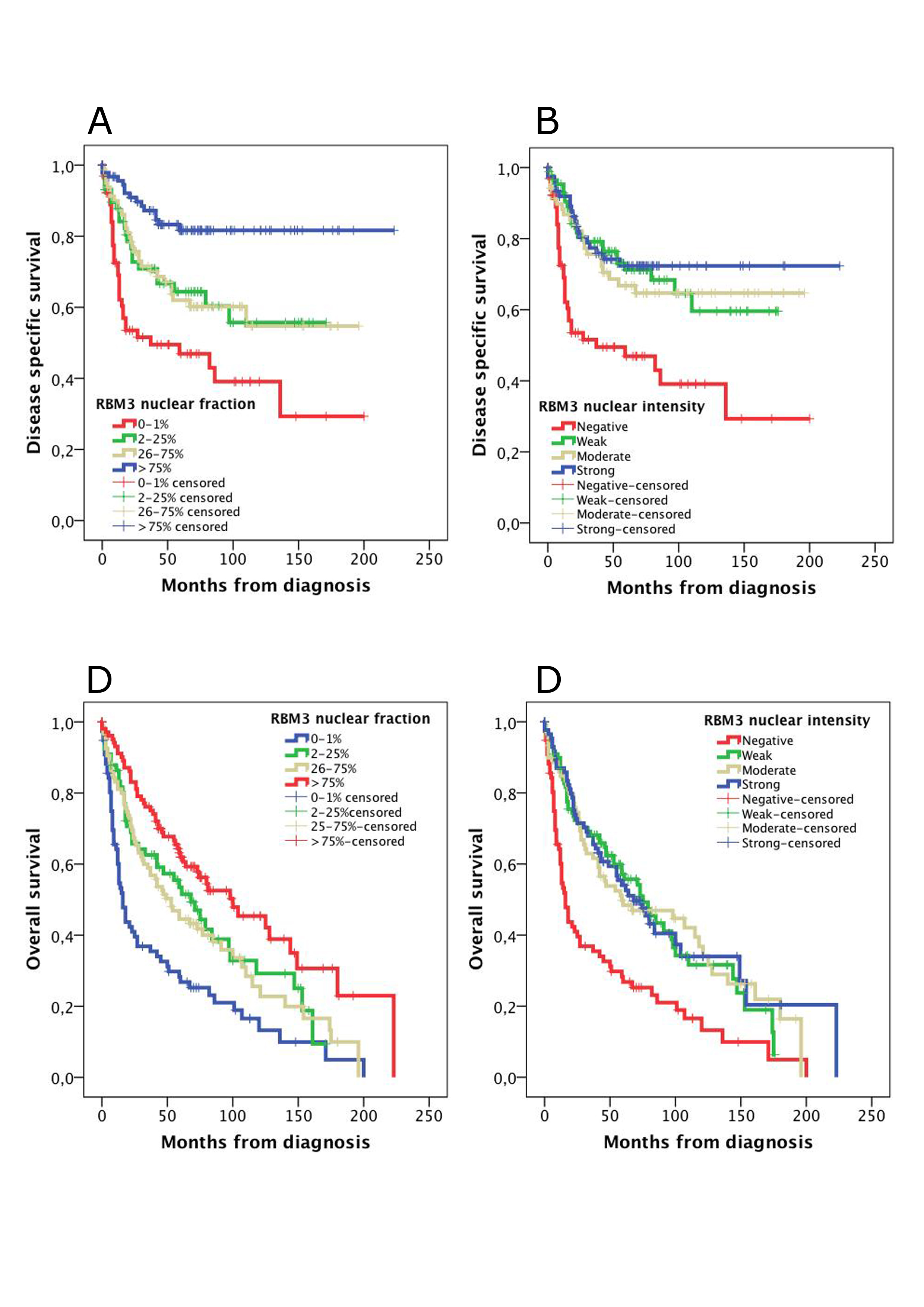

Supplement: Additional file 1 — Prognostic value of categories of nuclear fcation and intensity of RBM3 staining. Kaplan-Meier analysis of (A, B bladder cancer specific and (C, D) overall survival according to the nuclear fraction and intensity of RBM3 staining, respectively (logrank p over strata <0.001 for all). (JPEG 1141 kb) [file 1471-2490-13-17-S1.jpeg]
